# Beyond Cytoarchitectonics: The Internal and External Connectivity Structure of the Caudate Nucleus

**DOI:** 10.1371/journal.pone.0070141

**Published:** 2013-07-26

**Authors:** Sonja A. Kotz, Alfred Anwander, Hubertus Axer, Thomas R. Knösche

**Affiliations:** 1 Max Planck Institute for Human Cognitive and Brain Sciences, Leipzig, Germany; 2 School of Psychological Sciences, The University of Manchester, Manchester, United Kingdom; 3 Hans Berger Clinic for Neurology, Jena University Hospital, Friedrich-Schiller-University, Jena, Germany; Beijing Normal University, China

## Abstract

While there is ample evidence on the functional and connectional differentiation of the caudate nucleus (CN), less is known about its potential microstructural subdivisions. However, this latter aspect is critical to the local information processing capabilities of the tissue. We applied diffusion MRI, a non-invasive *in vivo* method that has great potential for the exploration of the brain structure-behavior relationship, in order to characterize the local fiber structure in gray matter of the CN. We report novel evidence of a functionally meaningful structural tri-partition along the anterior-posterior axis of this region. The connectivity of the CN subregions is in line with connectivity evidence from earlier invasive studies in animal models. In addition, histological validation using polarized light imaging (PLI) confirms these results, corroborating the notion that cortico-subcortico-cortical loops involve microstructurally differentiated regions in the caudate nucleus. Methodologically speaking, the comparison with advanced analysis of diffusion MRI shows that diffusion tensor imaging (DTI) yields a simplified view of the CN fiber architecture which is refined by advanced high angular resolution imaging methods.

## Introduction

**Figure 1:**
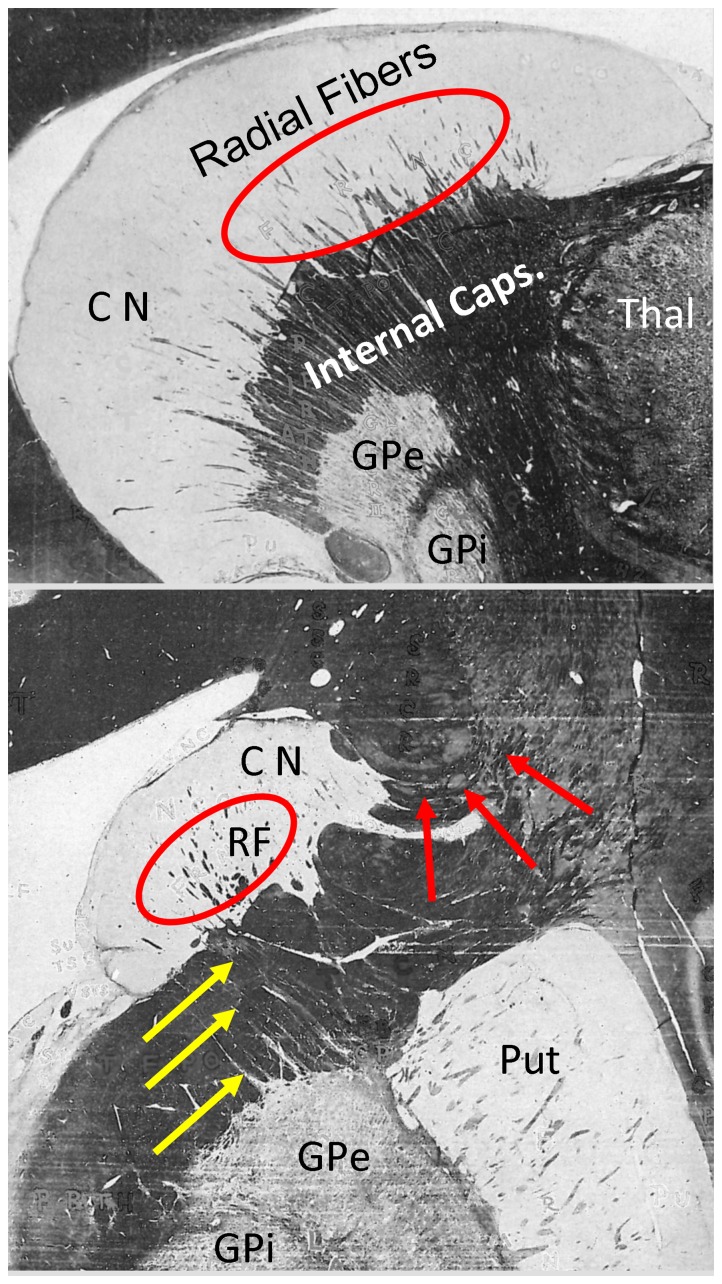
Sagittal (top) and coronal (bottom) myelin stained sections in a human cadaver brain (adapted from Riley, 1943). Radial fibers (RF) connecting the caudate nucleus (CN, circles) and the external globus pallidus (GPe) are visible (yellow arrows). In the right panel, connections from the CN to the neocortex are highlighted by red arrows. Anatomical structures are labeled: Put = putamen, GPi = internal globus pallidus, Thal = thalamus.

As part of the striatum, the caudate nucleus (CN) is functionally related to a variety of higher cognitive functions [1] and their inhibition [2]. More specifically, the CN seems to receive information from multiple cortical regions and transmits it via the globus pallidus (GP) and the thalamus back to the neocortex [for a meta-analysis, see 3] This view is corroborated by neuroanatomical evidence, for example, tracing studies for a review, see [4] and myelin stain evidence [5]; see Fig. 1. Very early studies of fiber degeneration as a consequence of cortical lesions in monkeys showed that cortico-striatal connections are organized in longitudinal (from head to tail) as well as transversal directions [6]–[9]. In short, these studies suggest that each part of the cortex projects to the nearest region of the striatum. However, later autoradiographic results questioned this proximity principle as certain cortical areas have target areas in the CN that spread along its entire length and are restricted to circumscribed areas in the coronal plane [10]–[12].

Such rich and diverse functional and anatomical connectivity patterns suggest the existence of distinguishable and potentially functionally relevant anatomical subunits in the CN that should be characterized by specific local microstructural traits, such as cytoarchitecture, local wiring, distribution of neurotransmitters and receptors, etc., and/or specific long-range connectivity patterns. Notably, the anatomical subdivision of the thalamus and other structures into nuclei and sub-nuclei has been mainly based on cytoarchitectonic and myeloarchitectonic traits. It seems that the available methods do not yield a similar result for the striatum see, e.g., [13], [14], except for very local distinctions between neurochemically defined patch/matrix areas [15]–[16]. Based on PET evidence, there is an established functional subdivision of the striatum into associative, limbic, and sensorimotor parts [17]. While the limbic part mainly comprises the ventral striatum, putamen and caudate nucleus are subdivided into a pre-commisural associative and a post-commisural sensorimotor part. However, due to the limited spatial resolution of the method, the precise boundaries remain unclear. On the other hand, neurochemical evidence [18]–[19] identifies a dorsal-ventral subdivision of the striatum into limbic, associative and sensorimotor parts.

Intrinsic anatomical connectivity, based on coherent fibers running within the gray matter as well as their respective long-range connections should form potent structural criteria for the definition of potentially functionally relevant subareas. Recently, diffusion MRI (dMRI) has developed into a tool for the noninvasive estimation of local fiber direction [20] and the reconstruction of fiber trajectories [21]. Its usefulness to the estimation of long-range white matter fiber trajectories and the exploitation of this for connectivity-based cortex parcellation has been demonstrated [22]–[24]. Previous studies of the basal ganglia suggest a segregated pattern of cortico-striatal connectivity [25]–[27]. Similar results have been found for the thalamus [28]. Moreover, and most importantly in the context of the current work, dMRI has been shown to differentiate areas in gray matter structures such as the thalamus and the amygdala, on the basis of the local fiber architecture [29]–[30]. Such parcellation has two major advantages that make it a useful complement to other methods of structural differentiation: (1) it is sensitive to the structure of local cortical circuits and hence, presumably, to the processing capabilities of the tissue, and (2) it can be applied non-invasively and *in vivo* and is therefore useful for the investigation of the structure-function relationship.

In the current work we use dMRI to infer on the internal structure and global connectivity of different sections of the CN and, most importantly, the relationship between the two factors. In particular, we demonstrate that dMRI allows non-invasive detection of sub-structure boundaries in the CN. We further demonstrate the long-range connectivity of the caudate nucleus, both in terms of its intrinsic connectivity and its cortical connectivity patterns and map these patterns to the sub-structure boundaries. In particular, the intrinsic microstructure proves plausible in light of additional polarized light imaging (PLI) findings. PLI is a new anatomical method to automatically estimate 3D fiber orientation in gross histological sections of the human brain [31]. It generates maps of vectors representing the main fiber orientation in every point of the brain section in high magnification. To our knowledge, this is the first account of a complete characterization of the caudate nucleus by *in vivo* dMRI accounting for microstructural subdivision, intrinsic, and long-range connectivity.

## Methods

We measured diffusion- and T1-weighted MR images in 13 (7 male) healthy, right-handed participants (mean age: 25±3 yrs) on a whole-body 3 Tesla Trio Siemens MR scanner (Siemens Healthcare, Erlangen, Germany) equipped with an eight-channel head array coil. This study was approved by the ethics committee of the University of Leipzig. All individuals in this study gave their informed consent for data collection, use and publication. Diffusion-weighted images were acquired with twice-refocused spin echo echo-planar-imaging sequence [32], TE = 100 ms, TR = 12 s, GRAPPA [33] acceleration factor 2, 128×128 image matrix, FOV = 220×220 mm^2^, providing 60 diffusion-encoding gradient directions with a b-value of 1000 s/mm^2^. Seven images without any diffusion weighting were obtained: one at the beginning of the scanning sequence and one after each block of 10 diffusion-weighted images as anatomical reference for offline motion correction. The interleaved measurement of 72 axial slices with 1.7 mm thickness (no gap) covered the entire brain. Random noise in the data was reduced by averaging 3 acquisitions resulting in a total acquisition time of about 45 minutes.

The T1-weighted structural scans were used for skull-stripping, and the brain images were then co-registered into Talairach space [34]. The CN was automatically segmented using Freesurfer [35]; https://surfer.nmr.mgh.harvard.edu/. The CN tail was not included in the segmentation due to the small cross-section area and the associated partial volume effects in diffusion imaging. The 21 images without diffusion weighting (seven b0 images for each of the three repetitions) distributed in the whole sequence were used to estimate motion correction parameters using rigid-body transformations [36], implemented in FSL (http://www.fmrib.ox.ac.uk/fsl). Motion correction for the 180 diffusion-weighted images was combined with a global registration to the T1 anatomy computed with the same method. The gradient direction for each volume was corrected using the rotation parameters. The registered images were linearly interpolated to the new reference frame with an isotropic voxel resolution of 1 mm and the three corresponding acquisitions and gradient directions were averaged. For each voxel, a diffusion tensor was computed from the images (DTI). Finally, local fiber orientation density functions (fODF) were computed using spherical deconvolution implemented in MRtrix http://www.brain.org.au/software/mrtrix; [37]. Additionally, all individual dMRI images were non-linearly registered [38] to the most typical brain within the group of participants. The most typical brain was defined as the one that required the least cumulative deformation when non-linearly registered to all other brains [39]. From this, group average diffusion tensor images were computed.

In order to compare the principal cell and myeloarchitectural structure of the caudate gray matter as evidenced by dMRI, we utilized polarized light imaging (PLI) on sagittal slices of the caudate nuclei of two human cadaver brains (one female, 65 years, one male, 67 years), who donated their bodies for anatomical study. This method has been described in detail elsewhere [31], [40]–[41]. In short, PLI is based on the birefringent properties of the myelin sheaths. The orientation of the nerve fibers in relation to the filter combination causes changes of light transmission through gross histological sections of the human brain under rotation of a polarization filter combination. This way, estimates of fiber direction and inclination are calculated at every point of the imaged section.

Sagittal slabs through the basal ganglia were serially sectioned with a cryostat microtome (CM3050 S, Leica Microsystems, Bensheim, Germany) with a thickness of 100 µm. Two sets of nine images each were recorded for each section, covering: (1) 90° separated by 10° rotations of the filters using two perpendicularly oriented polarization filters only, and (2) 180° separated by 20° rotations of the filters additionally using a quarter-wave plate (Axiocam HR, Carl Zeiss, Göttingen, Germany: basic resolution 1300×1030 pixel). Sinusoids were fitted to the nine intensity values at each pixel to recover the angle of fiber direction (in-plane orientation) and the angle of inclination (out-of-plane orientation). The magnification of the system was such that it resulted in pixels with dimension of 64×64 µm.

### Defining Internal Boundaries Based on Tissue Anisotropy

**Figure 2:**
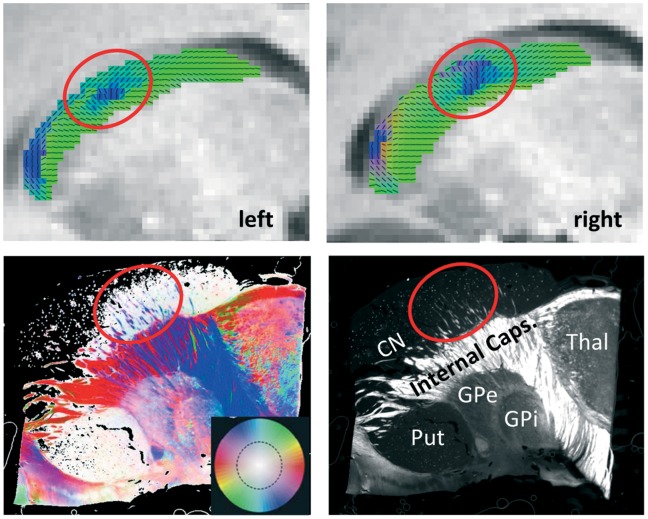
Direction-coded color images of the main diffusion direction derived from diffusion tensor imaging (DTI; blue: inferior-superior; red: left-right; green: posterior-anterior), averaged across participants, superimposed on sagittal anatomical slices of the typical subject (in Talairach space) of the left and right caudate nucleus (CN; see upper panels). In a sagittal view, the radial fibers (RF) of the CN (lower panels) can be seen as inferior-superior fiber bundles that connect the CN with the globus pallidus (GP) crossing the internal capsule (IC). Lower left: Fiber orientation map from polarized light imaging (PLI) of a sagittal section through the CN comparable to the section shown with DTI. Fiber directions within the image plane are color-coded and fiber inclination is saturation-coded (see color circle in the inset). Lower right: Maximum intensity map of the same section, which shows the maximum intensity of the polarization sequence and encodes the inclination of the fiber directions. White pixels mark myelinated fibers (dependent on their inclination, the steeper the darker), while dark pixels denote unmyelinated or perpendicular structures. Anatomical structures are labeled: CN = caudate nucleus, Put = putamen, GPe = external globus pallidus, GPi = internal globus pallidus, Thal = thalamus. Note that there is high coherence between the RFs in the CN, as visualized with PLI, and the fiber directions seen in DTI (circles). All individual subjects show similar patterns, see Figure S1.

In order to visualize differences in the anisotropic tissue structure in the CN, we mapped the principal diffusion direction as red-green-blue color code (Fig. 2) superimposed onto T1 images.

For quantitative analysis, we approximated the longitudinal axis of the CN by a spline fitted into all CN voxels using the angle with respect to the anterior commissure as a parameter. The spline was sampled equidistantly (on the parameter axis) at 45 points. Then all caudate voxels were projected onto these spline sample points and the angle of the principal tensor direction with the tangent of the spline was computed and averaged for each spline sample point. This *radiality index* was plotted against the distance along the spline. Prominent changes between predominantly radial and predominantly tangential anisotropy were used as markers for presumable microstructural boundaries (see Results).

### Charting the Connectivity of the Caudate

To chart the intrinsic and extrinsic connectivity of the CN we applied streamline tractography based on local fODF estimation MRTrix; [37]. We used the segmented volume of the CN as a seed region and seeded 500 streamlines at randomly selected positions (curvature radius 2 mm, minimum streamline length 30 mm, fODF amplitude cutoff 0.05). In order to combine the connectivity profiles of the individual subjects, the T1 images were registered to one typical subject using affine transformation implemented in FSL (*flirt*, 12 degrees of freedom). The transformation matrices were then applied to the points of the streamlines of the individual subjects using a purpose-written MatLab script. Then, the transformed streamlines were combined to form a cumulative connectivity profile. This profile was then subdivided into a number of bundles manually by placing inclusion and exclusion masks.

## Results

**Figure 3:**
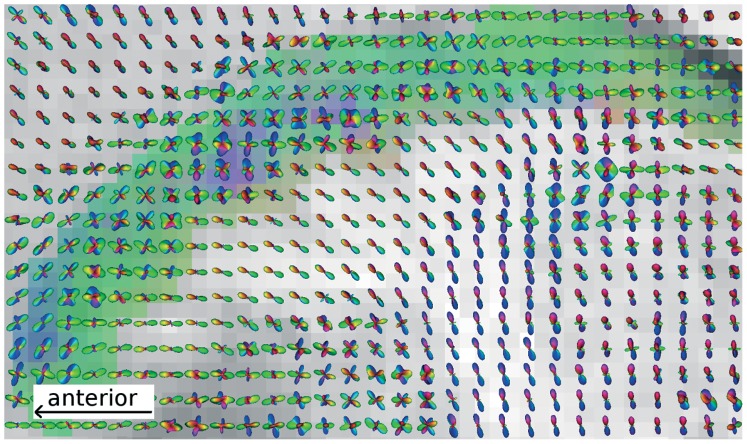
Complex fiber orientation structure visualized as orientation density functions overlaid to the direction-coded color image derived from diffusion tensor imaging (DTI). This sagittal image of a representative participant shows that the principal diffusion directions as defined by the diffusion tensors represent subtle but consistent imbalances within the complex fiber orientation profile of the caudate nucleus (CN) gray matter. While fibers running along the axis of the CN outweigh the fibers entering from the adjacent white matter of the internal capsule (IC) in the posterior portion, in the anterior portion, it seems that fibers entering from the IC dominate the diffusion profile. The middle portion with inferior-superior principal directions (blue area) is characterized by additional fibers restricted to the caudate and pointing in the direction of the external globus pallidus (GPe). All individual subjects show similar patterns, see Figure S2.

With dMRI we are able to show anisotropic diffusion in the CN (mean fractional anisotropy (FA) ranges from 0.15 to 0.22 across participants) with consistent orientation patterns across all participants (see Fig. 2, upper panel, for averaged DTI images). The main orientation throughout the CN was posterior-anterior (head and body). All participants showed a cluster of voxels with supero-inferior orientation in the middle portion of the CN body. This region also partitions the CN into an anterior, middle, and posterior portion. The reconstruction of the fiber orientation density functions (fODF) using the spherical deconvolution technique [37] yields a more detailed picture of the fiber architecture of the caudate and adjacent areas (see Fig. 3). It shows that the principal diffusion direction, as defined by the diffusion tensor, represents subtle but consistent imbalances within the complex fiber orientation profile of the CN gray matter. The fODF reveals that in the entire CN, more than one fiber direction exists. In particular, there seems to be one fiber population running along the axis of the CN as well as various populations crossing this bunFdle, including horizontal fibers connecting the caudate head to other brain structures via the IC and, most prominently, radial fibers running between the GP and the middle portion of the CN, thereby causing the aforementioned primarily inferior-superior direction observed in the DTI images. The latter fibers have also been referred to as “Wilson’s pencils” [42] or “fasciculi radiales nuclei caudatae” (Riley, 1943; see Fig. 1). In the following, we refer to these fibers as radial fibers.

Inspection of sagittal PLI images (see Fig. 2, lower panels) suggests that the inferior-superior fiber orientation in the middle CN portion is related to fiber bundles that run between the CN and the external GP (GPe) through the IC. This is consistent with the radial fibers described above. The myelinated radial fibers, organized as bundles in the caudate nucleus, can be distinguished in great detail using PLI as shown in the fiber orientation maps. The radial fibers cross the anterior part of the internal capsule and connect the GPe. In the PLI images they are the only distinct fiber bundles in the caudate nucleus, while the main part of the caudate is low in myelin, manifested as dark areas in the maximum intensity maps (see legend Fig. 2).

**Figure 4:**
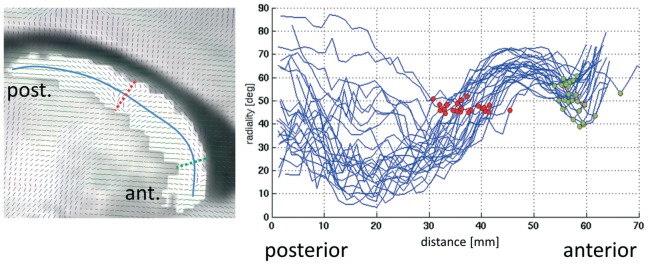
Segmentation of the caudate nucleus according to the principal diffusion orientation. Left: sagittal slice through one example caudate nucleus, showing the principal diffusion directions (little dashes). The blue curve represents the longitudinal spline (see Methods section) along which the directions are sampled. As a general pattern, the diffusion direction is largely tangential (with respect to the longitudinal spline) in the caudal part of the caudate nucleus. At some point the orientation turns into predominantly radial (red dashed line at approx. 45°). Further anterior, there is again a section with tangential orientation (green dashed line at maximally tangential position), and towards the anterior tip they become radial again. Right: Radiality index (angle of principal diffusion direction with longitudinal spline) plotted against distance along the longitudinal spline, for all subjects and hemispheres. The red discs denote the 45° points (equivalent to red dashed line in left panel), while the green discs indicate the local minimum of the radiality index (i.e. the points with maximally tangential orientation; equivalent to green dashed line in left panel).

Visual inspection of the local principal diffusion orientation (Fig. 2) makes apparent the existence of a zone with primarily vertical fibers (Wilson’s pencils) in the mid CN, suggesting a subdivision into three major sections along the longitudinal axis of the CN. Accordingly, we identified two consistent microstructural boundaries along the main axis of the CN: one approximately in the middle of the caudate body, where the main fiber orientation switches from predominantly tangential (posterior part) to mainly radial (anterior part), and one close to the caudate head, where the mainly radial fibers are interrupted by a narrow zone with more tangential orientation (see Fig. 4). For the former point, we identified, in the central part of the CN, the point where the mean angle between the midline and the local diffusion direction over all subjects was 45 degrees. For the latter point, we identified the local minimum just near the caudate’s head (see Fig. 4). Then, for the former point, we searched in each individual midline within a radius of 10 mm for the point, where the angle was closest to 45. For the latter point we searched in each individual midline, again within a radius of 10 mm, for the point with the lowest angle.

**Figure 5:**
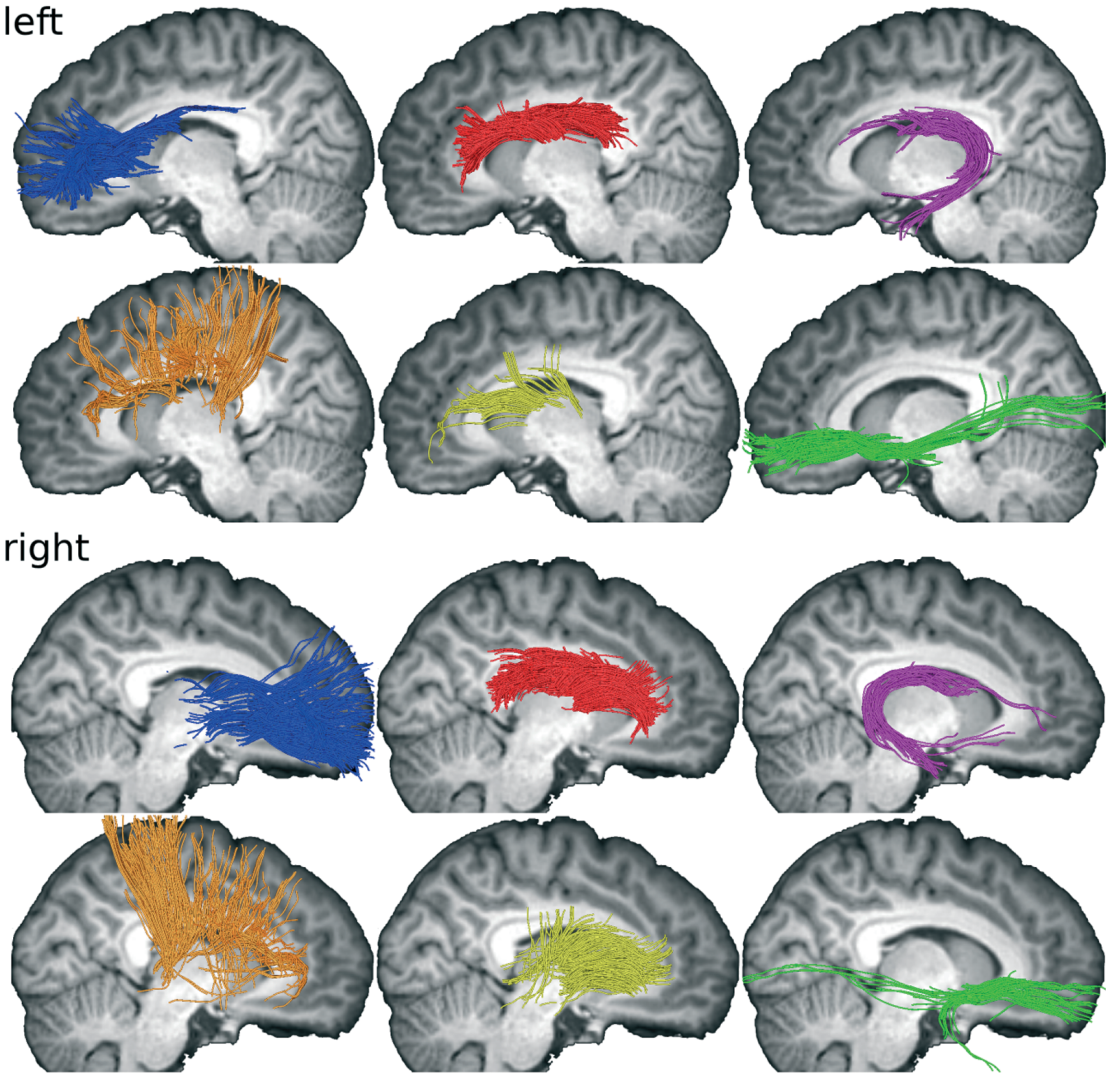
Major fiber populations connecting the CN as reconstructed using streamline tractography based on spherical deconvolution. Fibers from all 13 subjects are overlaid to T1 slices of on subject. Color coding: Longitudinal fiber pathways running along the head and body of the CN (red); fiber pathways following the tail of the CN (purple); fiber pathways leaving the head of the CN towards the frontal pole (blue); a thin ventral bundle of fibers connecting the inferior CN head region with temporal and occipital cortices (green); several fiber pathways running in parallel to the radiating fibers in the internal capsule appear to penetrate the CN (orange); fiber pathways from the thalamus enter the mid portion of the CN (yellow).

**Figure 6:**
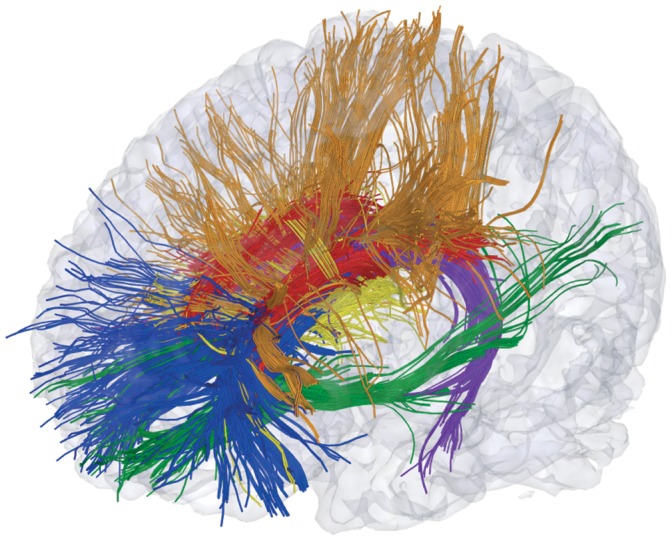
Three-dimensional rendering of the fiber populations of the caudate (see Fig. 5 for details). For an interactive version of this figure see Figure S3.

In a next step, anatomical connectivity of the CN was explored using tractography (Fig. 5). In order to facilitate visual assessment of the connectivity, several major fiber populations connecting the CN were identified according to their major connections outside the CN by placing inclusion/exclusion masks. First, longitudinal fiber pathways are running along the head and body of the CN without reaching distant sites (Fig. 5 red fiber pathways; exclusion masks in prefrontal cortex, temporal lobe including tail of CN, dorsal somatosensory/motor/premotor cortex, thalamus), following the tail of the CN (Fig. 5 purple fiber pathways; inclusion mask in tail of CN), and connecting the head of the CN with the frontal pole (Fig. 5 blue fiber pathways; inclusion mask in prefrontal cortex anterior of BA 8 [according to Talairach coordinates], exclusion mask in temporal lobe). Second, there is a thin ventral bundle of fibers connecting the inferior CN head region with temporal and occipital cortices (Fig. 5 green fiber pathways; two AND-combined inclusion masks in prefrontal cortex and temporal lobe). Third, several fiber pathways running in parallel to the radiating fibers in the internal capsule appear to penetrate the CN (Fig. 5 orange fiber pathways; inclusion mask comprising dorsal somatosensory/motor/premotor cortex and BA 8). Lastly, fiber pathways connect the thalamus with the mid portion of the CN (Fig. 5 yellow fiber pathways; inclusion mask in thalamus). All masks were determined manually using the T1 anatomy and the reference brain and a brain atlas [43]. An interactive 3D representation of the data is provided in Figure 6 and Figure S3.

**Figure 7:**
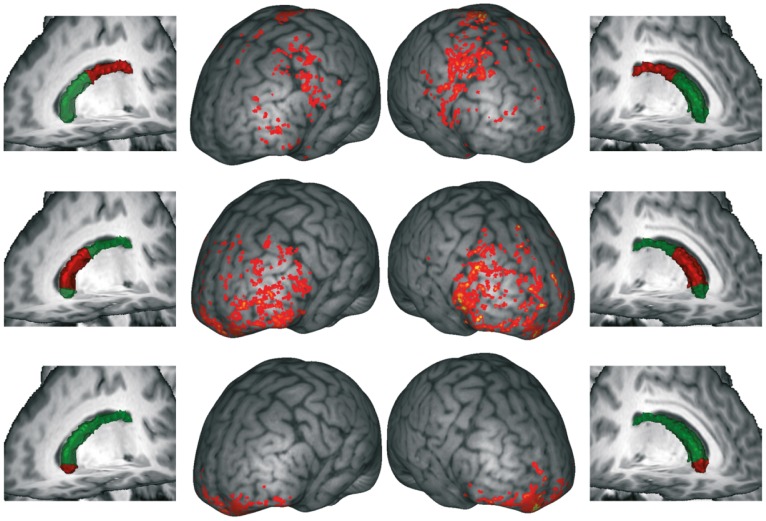
Cortical targets of fibers originating in the caudate subregions. Center columns: Cumulative projection sites from all subjects, overlaid to T1 image of one subject (brain surface distance map). Outer columns: Surfaces of caudate nuclei (green) and respective subregion (red) for one subject.

In order to obtain a more detailed account of the CN connectivity, and to link it with its internal structure, we mapped the cortical target areas of the three sections of the CN as defined by the internal structure boundaries (see above). In Fig. 7 we show that most of the connections are with the frontal lobe. The caudal part of the CN body (with mainly tangential fiber orientations) features connectivity to the precentral gyrus and in the inferior frontal gyrus. The more radially oriented middle part predominantly connects to prefrontal cortex. Finally, the rostral most section connects to orbitofrontal and frontopolar regions. Note that the temporal connections described above (Fig. 5) are not visible as cortical projections in Figure 7 as they could not be followed all the way to their cortical targets by the tractography algorithm.

## Discussion

### Structural and Functional Implications

There is ample evidence on the functional differentiation of the caudate nucleus as well as of a highly organized arrangement of cortical-striatal connectivity. However, neurochemical structural evidence does not completely agree with such functional subdivision. Moreover, there is little to no evidence on potential subdivisions of this structure based on cyto- or myeloarchitecture that would potentially map onto such functional diversity. However, traditional anatomical parcellation methods based on cytoarchitectonics may only miss ‘functional’ organization based on local fiber architecture [13]. This is surprising in light of early myelin stain evidence suggesting that structural differentiation within the caudate nucleus (CN) may be based on fiber architecture. For example, Riley [5]; see Fig. 1] showed radial fibers penetrating the CN that seem to continue to various white matter fiber tracts and the globus pallidus (GP). These fibers seem to be identical to radially oriented efferent fiber bundles (Wilson’s Pencils) running between the striatum and the GP, and continuing to the substantia nigra [see 44]. Application of polarized light imaging (PLI) confirms the existence of a middle section of the CN with prominent radial fibers [45]. The PLI images suggest that these radial fibers connect ventrally to the external globus pallidus (GPe).

Based on this early neuroanatomical evidence we aimed to obtain *in vivo* non-invasive characterization of fiber structure in gray matter using dMRI. We report novel evidence of a meaningful neuroanatomical subdivision along the longitudinal axis of the CN based on the local fiber architecture of the tissue. These sections exhibit specific connectivity patterns to regions of the cortex, mainly in the frontal lobe. Some of these connections, in particular to the precentral gyrus, run in parallel to the radiating fibers of the IC. This is in line with evidence from myelin stains suggesting that fiber tracts leaving the CN join the inferior-superior fiber pathway of the IC (see Fig. 1). It is also in line with early evidence based on fiber degeneration due to cortical lesions, suggesting an arrangement of cortico-striatal connectivity along the longitudinal axis of the CN [e.g., 8]. Moreover, this subdivision also largely reflects the neurochemical results of Karachi and colleagues (2002), although there are some distinctions with respect to the dorsal and ventral parts of the caudate body that may have not been sensitively detected in our current methodological approach.

More specifically, our results reveal that the anterior section, with primarily anterior-posterior fiber directions, is connected with lateral and medial prefrontal cortices (BA 10, 11, 47), while the middle section, containing the radial fibers, features connections to the entire prefrontal cortex (Fig. 7). This is in strong agreement with functional connectivity findings [46], earlier tractography studies [26], [47]–[48], tracing results in the monkey [12], [49]–[50], and recently with clinical implications of striato-cortical functions [51]. The posterior portion of the caudate body seems to be connected to motor and premotor cortices (Fig. 7). On the one hand, earlier tractography evidence has revealed connections from the posterior caudate body to the SMA proper [47]–[48] and M1 [26]. On the other hand, looking at myelin stained sections through the posterior portion of the CN body [see 5] seems to suggest that no myelinated fibers connect this section with the IC. One possible explanation for this discrepancy is that fibers crossing the boundary of the posterior CN body are not myelinated, at least not in the vicinity of the CN.

A second prominent feature of the fiber architecture of the CN suggested by the present results is the existence of a fiber population running along the longitudinal axis of the CN. Interestingly there is no evidence of such fibers in the PLI and myelin stain images. Likewise there is no report in the literature of fibers running along the main axis of the CN. This indicates that they are most likely short, but coherently aligned, unmyelinated axons that connect adjacent sections of the CN. Hence, the fODF data seem to suggest that the different portions of the CN are connected by longitudinal fibers that may support supramodal (sensorimotor) integration of different cognitive functions of various levels of complexity. This supports the functional interpretation of the CN as a significant player in higher cognitive operations e.g., [1], [2]. This seems to be in agreement with some of the early fiber degeneration studies showing that lesions in a number of cortical areas lead to fiber degeneration along the entire CN, but strictly bounded to certain portions of the cross-sectional plane [11].

Thirdly, there seems to be a strong connection between the caudate head and the medial frontal pole as recently also described by Catani and colleagues [52] and Langen and colleagues [53]–[54] as fronto-striatal tracts.

Fourthly, there are also fiber pathways connecting the CN via the external/extreme capsule with the temporal lobe and partially with the occipital lobe. Similar patterns of anatomical connectivity have also been found in monkey tracer studies [12], [49]–[50].

### Methodological Implications

DTI was used to characterize the local fiber architecture in the CN. Although it is very powerful, this technique has two main caveats: (1) the limited spatial resolution involves the risk that adjacent and qualitatively different features are merged together, and (2) the approximation by a diffusions tensor does not allow the representation of complex fiber arrangements, such as crossings or branchings. Here, we used different strategies to assess the impact of these limitations on the conclusions drawn: PLI has a much higher resolution compared to DTI, while the computation of fiber orientation density functions (fODFs) reveals more details of the angular fiber distributions and is able to resolve crossings.

With respect to the latter, the fODF reveals that in the entire CN, there are complex configurations of crossing fiber bundles. Subtle but consistent imbalances between the volume fractions of the crossing fiber populations appear to be responsible for the principal direction boundaries in DTI used to delineate function-anatomical sections. In particular, this applies to the appearance of radial fibers in the middle portion of the CN, which are reflected by a predominantly inferior-superior diffusion orientation in both DTI and fODF. However, inspection of the fODF (Fig. 3) also reveals details about additional fiber populations (e.g., horizontal fibers entering the ventral posterior head of the CN from the IC), which may be important for a further subdivision of the CN. Our results show that DTI is sensitive to aspects of microstructure in gray matter, which may be functionally relevant because they are correlated to long-range connectivity. Comparison between tensor and fODF results suggests that in this case the interpretation with respect to the main diffusion direction is largely similar. In other words, tensors yield simplified, but correct information on the microstructure, and the fODF complements rather than contradicts these results. However, this cannot be taken for granted. For example, Leergard and colleagues [55] demonstrates that fiber directions estimated from the diffusion tensors can be outright wrong in areas with crossing fiber populations of similar volume fractions.

Both DTI and PLI reflect fiber orientation in brain tissue. The advantage of PLI as a histological validation method of DTI is its much higher resolution (64×64×100 µm in this study). However, while PLI histological processing has the potential for high-resolution 3D reconstruction of the human brain [41], it is a very elaborate and time-consuming method, and, moreover, is only feasible in post-mortem specimens. In contrast, DTI can be applied non-invasively in living humans. Therefore, PLI may be most effectively used as a validation method for DTI. Accordingly, in the current study, PLI of the CN was applied to validate DTI results from the same region. PLI allowed visualization of the radial fibers in the CN in detail. These structures are too small to be resolved in detail using DTI. Nevertheless, they cause a diffusion preference, which gives rise to a DTI correlate in many of the voxels. In this respect, PLI verified the DTI results and visualized the radial fibers as the respective anatomical correlate.

With respect to tractography it is important to note that this method only yields estimates of possible fiber pathways rather than actual fibers of fiber bundles. In other words, we cannot say from tractography alone to what extend for example the longitudinal fibers within the CN represent continuous axons or short segments of fibers (see argumentation above). Vice versa the separate reconstruction of fiber pathways, for example, the pathways in the caudate tail, in head and body of the caudate and those pathways leaving the caudate head towards the frontal pole, does not allow concluding that there are not axons running all the way from the caudate tail to the frontal pole. Furthermore, limited spatial and angular resolution may cause streamlines to “jump” between fiber bundles, in particular preferring the large long-distance fiber bundles. Hence, it is possible that some of the trajectories associated to a particular seed region in the CN actually belong to fibers that just pass that seed region. Increased spatial resolution might help to improve this situation [see, e.g., 56,57].Therefore, it is of utmost importance to integrate evidence from different methods in order to reach valid conclusions as we have done by considering both tractography and myelin staining in the case of the longitudinal fibers in the CN (see above).

## Conclusions

We show that DTI is a powerful method to reveal subdivisions in the CN that are based on local structural properties and correlate to global anatomical connectivity patterns. The method is non-invasive and therefore has great potential for the exploration of the relationship between brain structure and behavior. The results were qualitatively confirmed by PLI. The findings support the notion that cortico-subcortico-cortical loops [4] involve circumscribed regions in the caudate nucleus that differ in terms of their local tissue structure. The results further show that the anterior-posterior organization of cortico-striatal connections that was already suggested by early fiber degeneration studies in animals is paralleled by a longitudinal organization based on local tissue structure in humans. In terms of methodology, our findings demonstrate that diffusion tensor imaging (DTI) yields a simplified view of the CN fiber architecture, which is refined by the use of advanced high angular resolution imaging methods.

## Supporting Information

Figure S1 **Direction-coded color images of the main diffusion direction derived from diffusion tensor imaging (DTI; blue: inferior-superior; red: left-right; green: posterior-anterior) of all participants, superimposed on sagittal anatomical slices (in Talairach space) of the left and right caudate nuclei.** For each participant we show a central slice through the head and body of the CN. The representation shows only a central slice. For additional details see legend Figure 2.
(PDF)

Figure S2 **Complex fiber orientation structure visualized as orientation density functions overlaid to the direction-coded color image derived from diffusion tensor imaging (DTI).** This sagittal images of all participants shows that the principle diffusion directions as defined by the diffusion tensors represent subtle but consistent imbalances within the complex fiber orientation profile of the caudate nucleus (CN) gray matter. While fibers running along the axis of the CN outweigh the fibers entering from the adjacent white matter of the internal capsule (IC) in the posterior portion, in the anterior portion, it seems that fibers entering from the IC dominate the diffusion profile. The middle portion with inferior-superior principal directions (blue area) is characterized by additional fibers restricted to the caudate and pointing in the direction of the external globus pallidus (GPe).
(PDF)

Figure S3 **Three-dimensional interactive rendering of the fiber populations of the caudate (see Fig 5 for details).** Click into the Figure to activate the interface. Left mouse click permits rotation, wheel of zooming. The 3D model was integrated in the portable document format (PDF) using SimLab Composer (SimLab Soft., Amman, Jordan) and requires the use of a compatible PDF reader (e.g. Adobe Reader 9).
(PDF)
