# Supplementary material for: Beyond Cytoarchitectonics: The Internal and External Connectivity Structure of the Caudate Nucleus
Source: PLoS One. 2013 Jul 26;8(7):e70141. doi: 10.1371/journal.pone.0070141 (PMC3724823; doi:10.1371/journal.pone.0070141)
Supplement: Figure S1 — Direction-coded color images of the main diffusion direction derived from diffusion tensor imaging (DTI; blue: inferior-superior; red: left-right; green: posterior-anterior) of all participants, superimposed on sagittal anatomical slices (in Talairach space) of the left and right caudate nuclei. For each participant we show a central slice through the head and body of the CN. The representation shows only a central slice. For additional details see legend Figure 2. (PDF) [file pone.0070141.s001.pdf]

Beyond cytoarchitectonics: The internal and external connectivity structure of the caudate nucleus

Short title: Connectivity structure of the caudate nucleus

Sonja A. Kotz[1, *], Alfred Anwander[1], Hubertus Axer[2], and Thomas R. Knösche[1]

[1]Max Planck Institute for Human Cognitive and Brain Sciences, Leipzig, Germany

[2]Hans Berger Clinic for Neurology, Jena University Hospital, Friedrich-Schiller-University, Jena, Germany

Supplementary material includes:

2 Figures

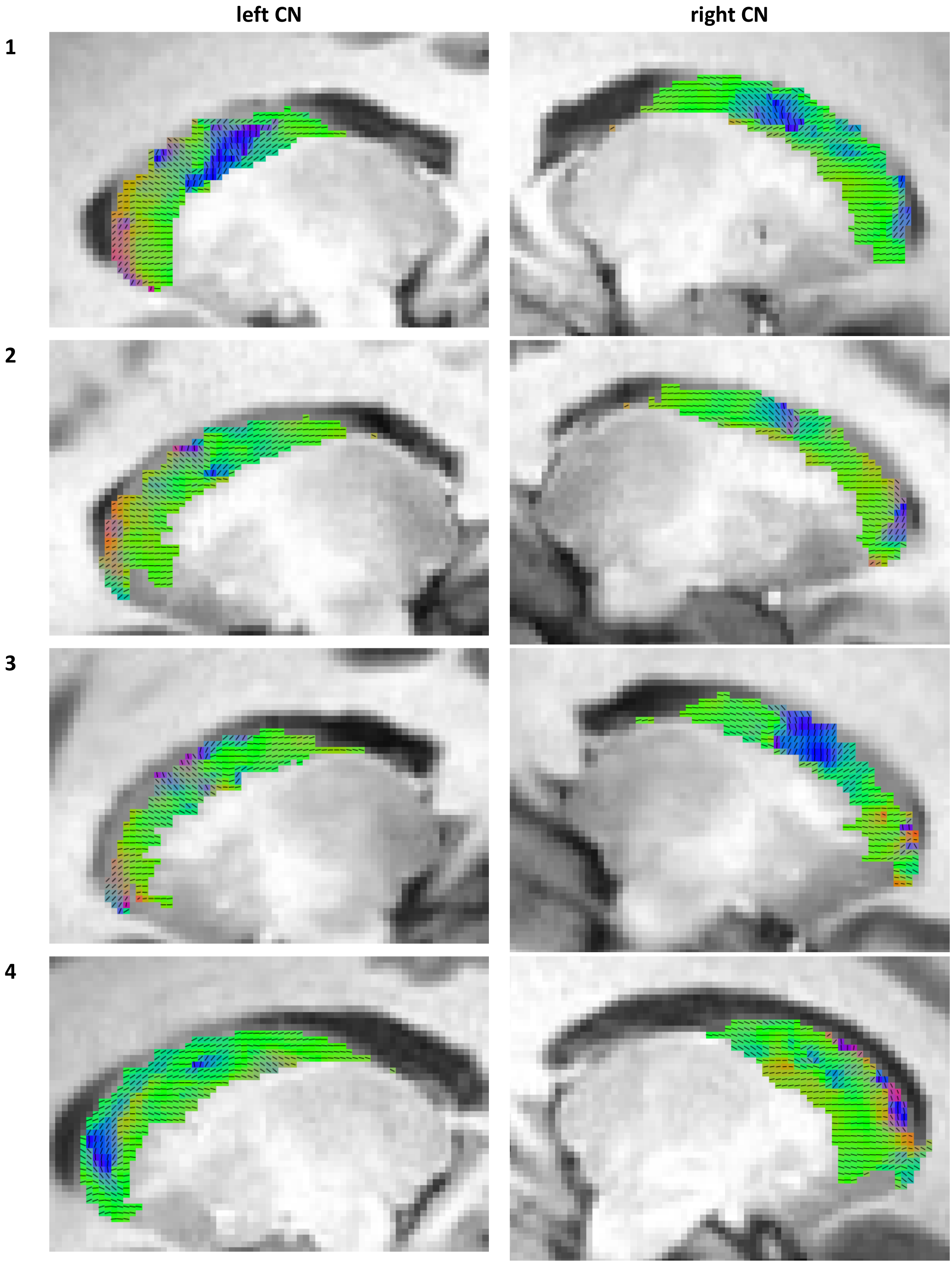

left CN
right CN
1
2
3
4

5

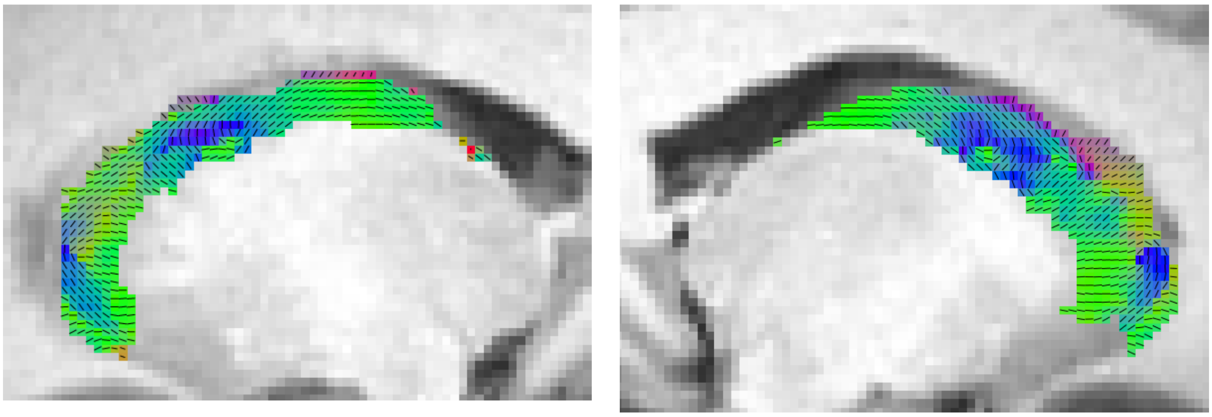

6

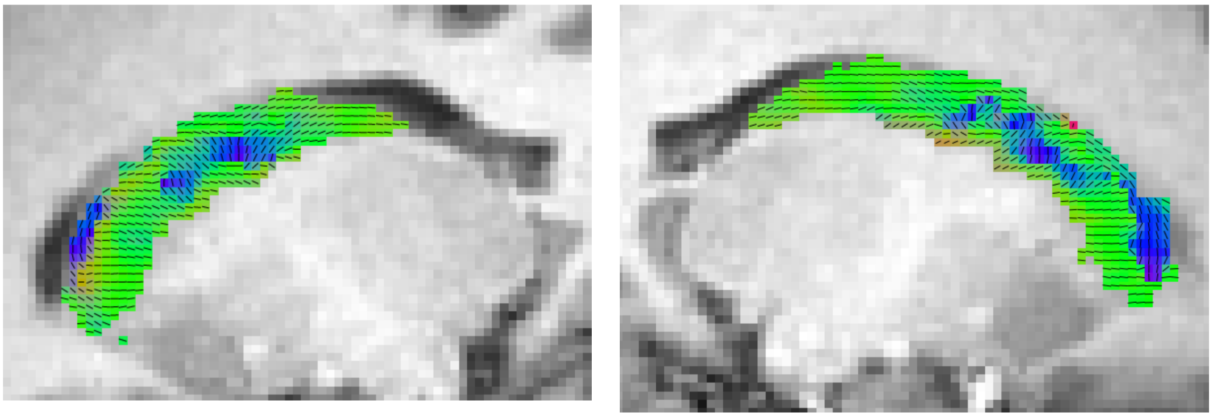

7

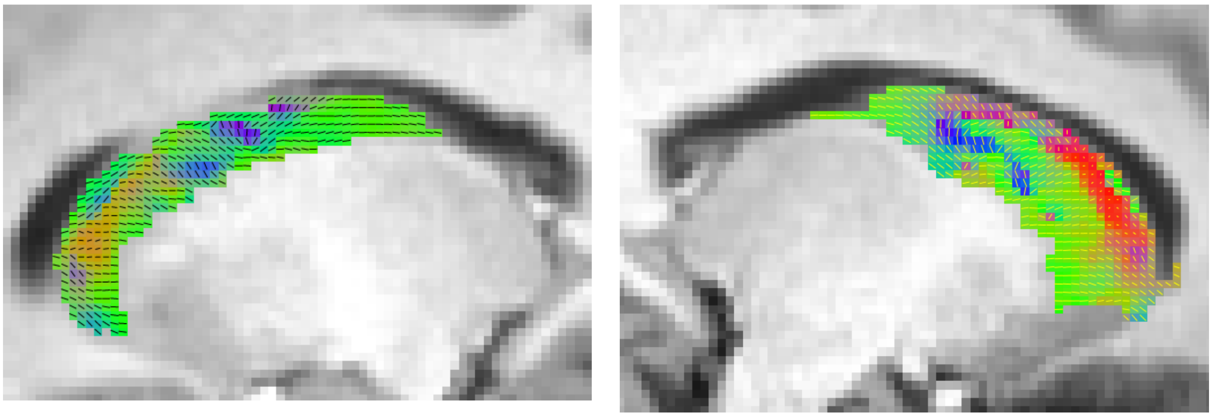

8

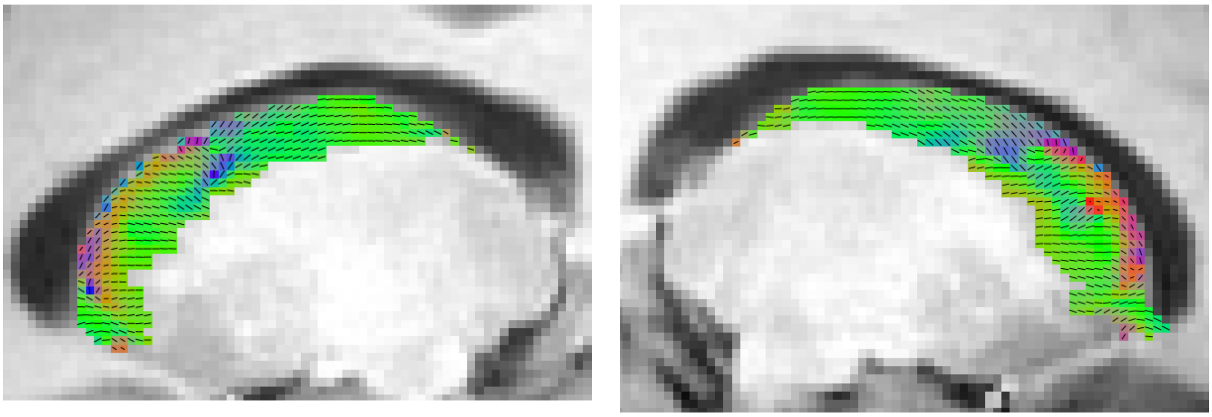

9

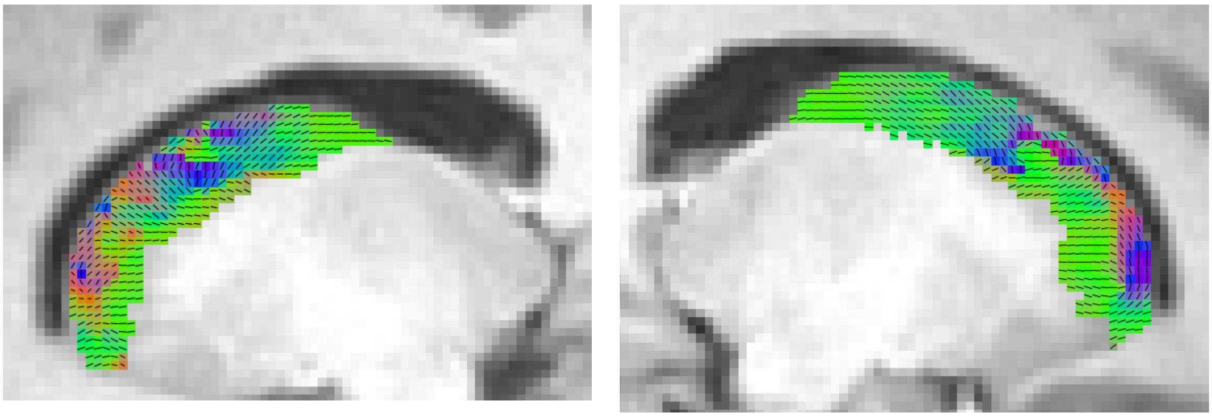

10

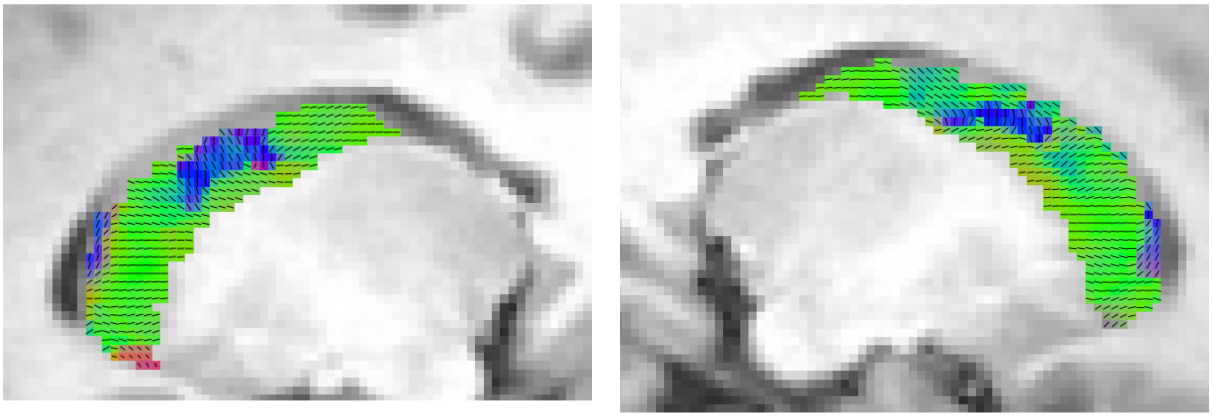

11

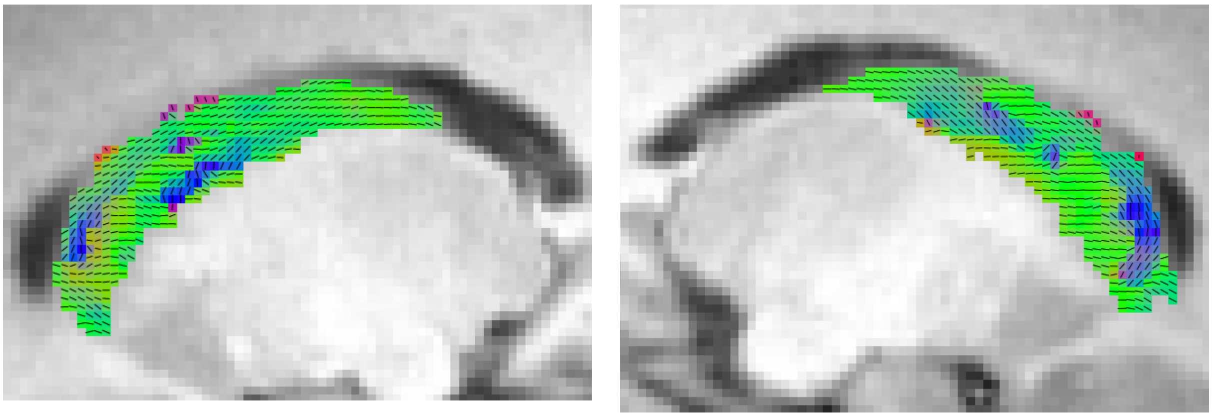

12

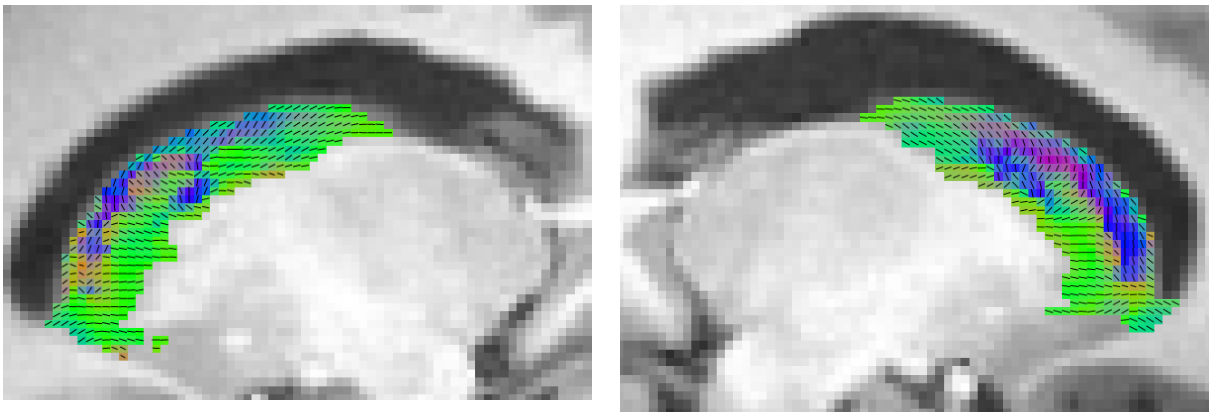

13

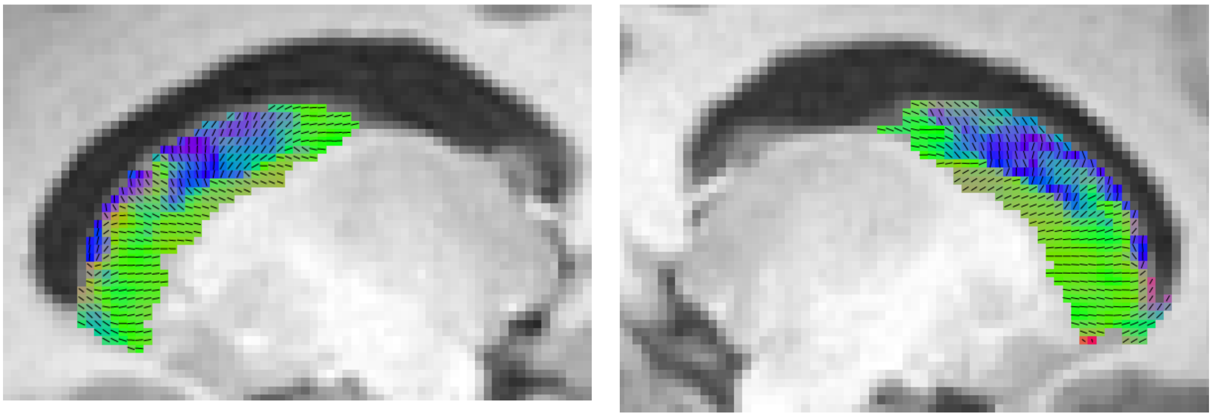

Figure S1: Direction-coded color images of the main diffusion direction derived from diffusion tensor imaging (DTI; blue: inferior-superior; red: left-right; green: posterior-anterior) of all participants, superimposed on sagittal anatomical slices (in Talairach space) of the left and right caudate nuclei. For each participant we show a central slice through the head and body of the CN. The representation shows only a central slice. For additional details see legend Figure 2.
