# Supplementary material for: Beyond Cytoarchitectonics: The Internal and External Connectivity Structure of the Caudate Nucleus
Source: PLoS One. 2013 Jul 26;8(7):e70141. doi: 10.1371/journal.pone.0070141 (PMC3724823; doi:10.1371/journal.pone.0070141)
Supplement: Figure S2 — Complex fiber orientation structure visualized as orientation density functions overlaid to the direction-coded color image derived from diffusion tensor imaging (DTI). This sagittal images of all participants shows that the principle diffusion directions as defined by the diffusion tensors represent subtle but consistent imbalances within the complex fiber orientation profile of the caudate nucleus (CN) gray matter. While fibers running along the axis of the CN outweigh the fibers entering from the adjacent white matter of the internal capsule (IC) in the posterior portion, in the anterior portion, it seems that fibers entering from the IC dominate the diffusion profile. The middle portion with inferior-superior principal directions (blue area) is characterized by additional fibers restricted to the caudate and pointing in the direction of the external globus pallidus (GPe). (PDF) [file pone.0070141.s002.pdf]

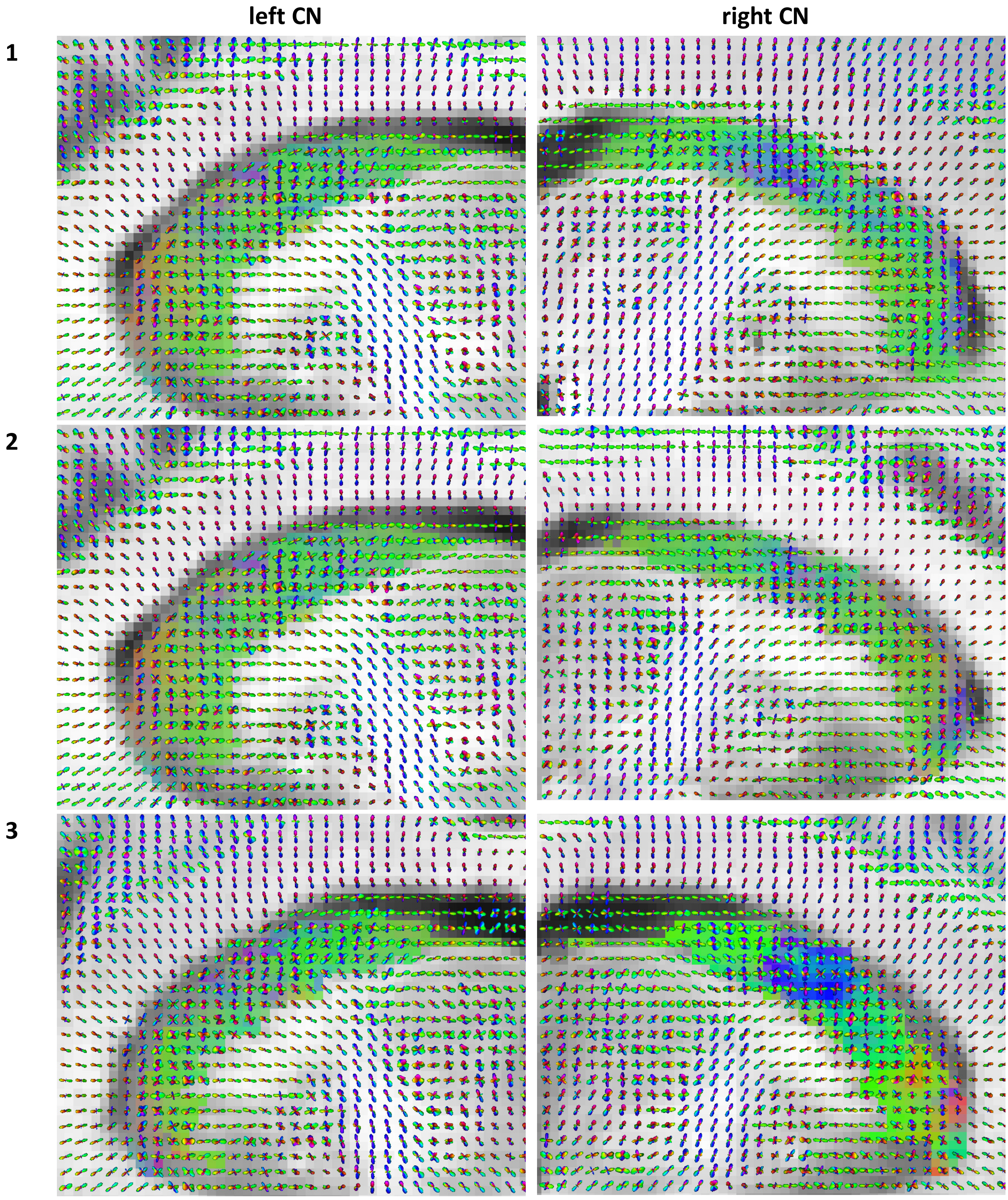
left CN
right CN
1
2
3

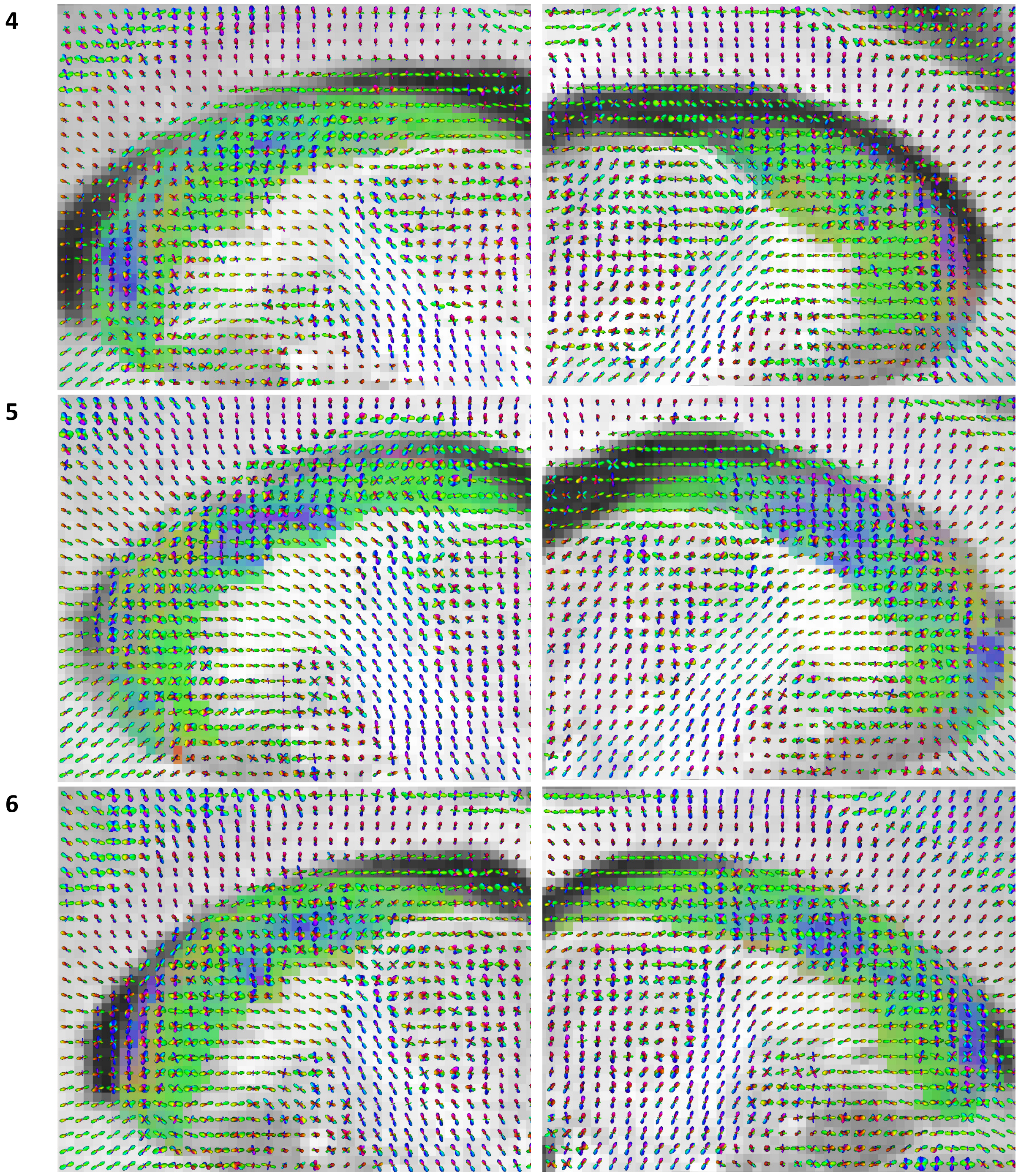
4
5
6

7

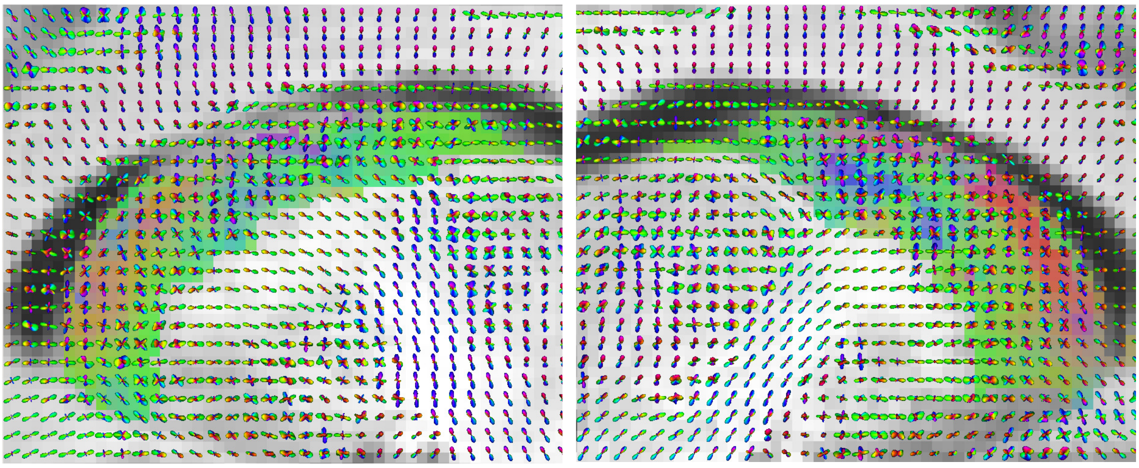

8

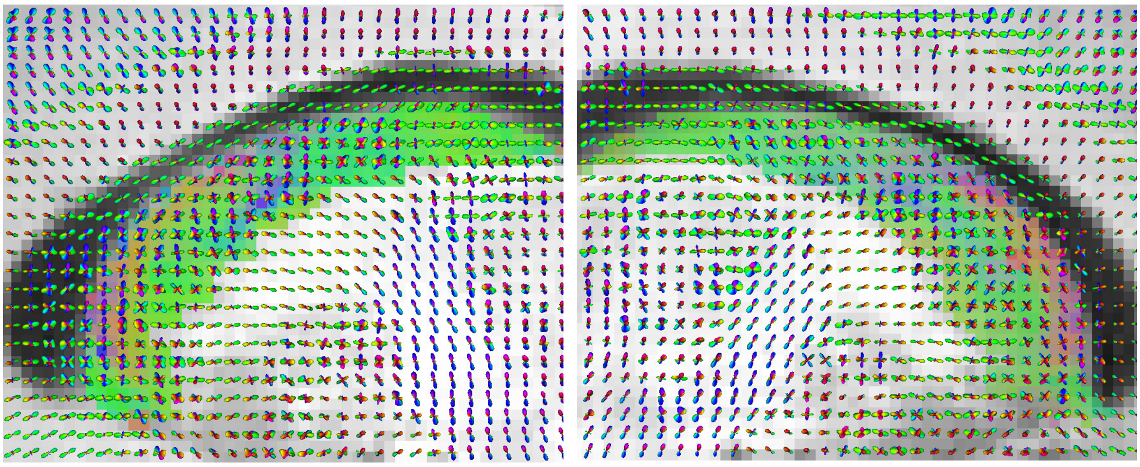

9

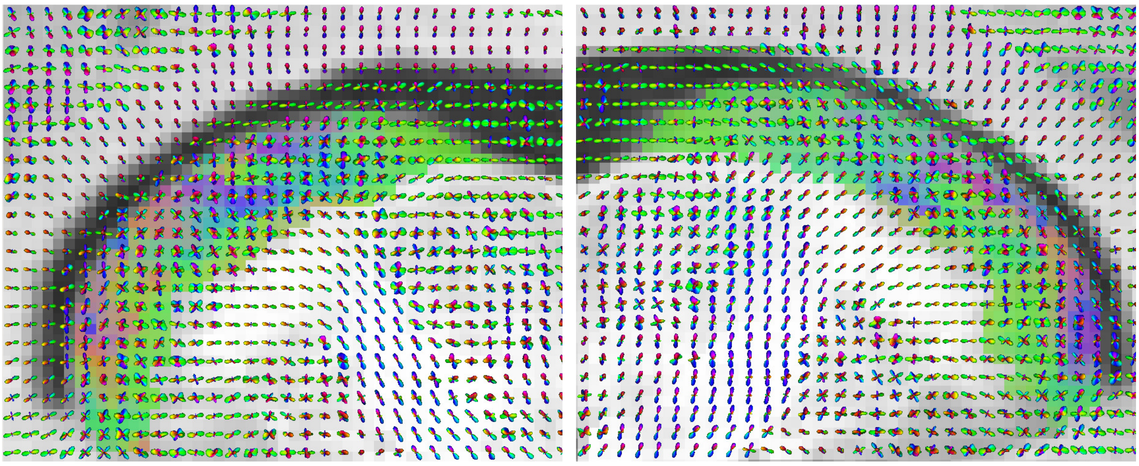

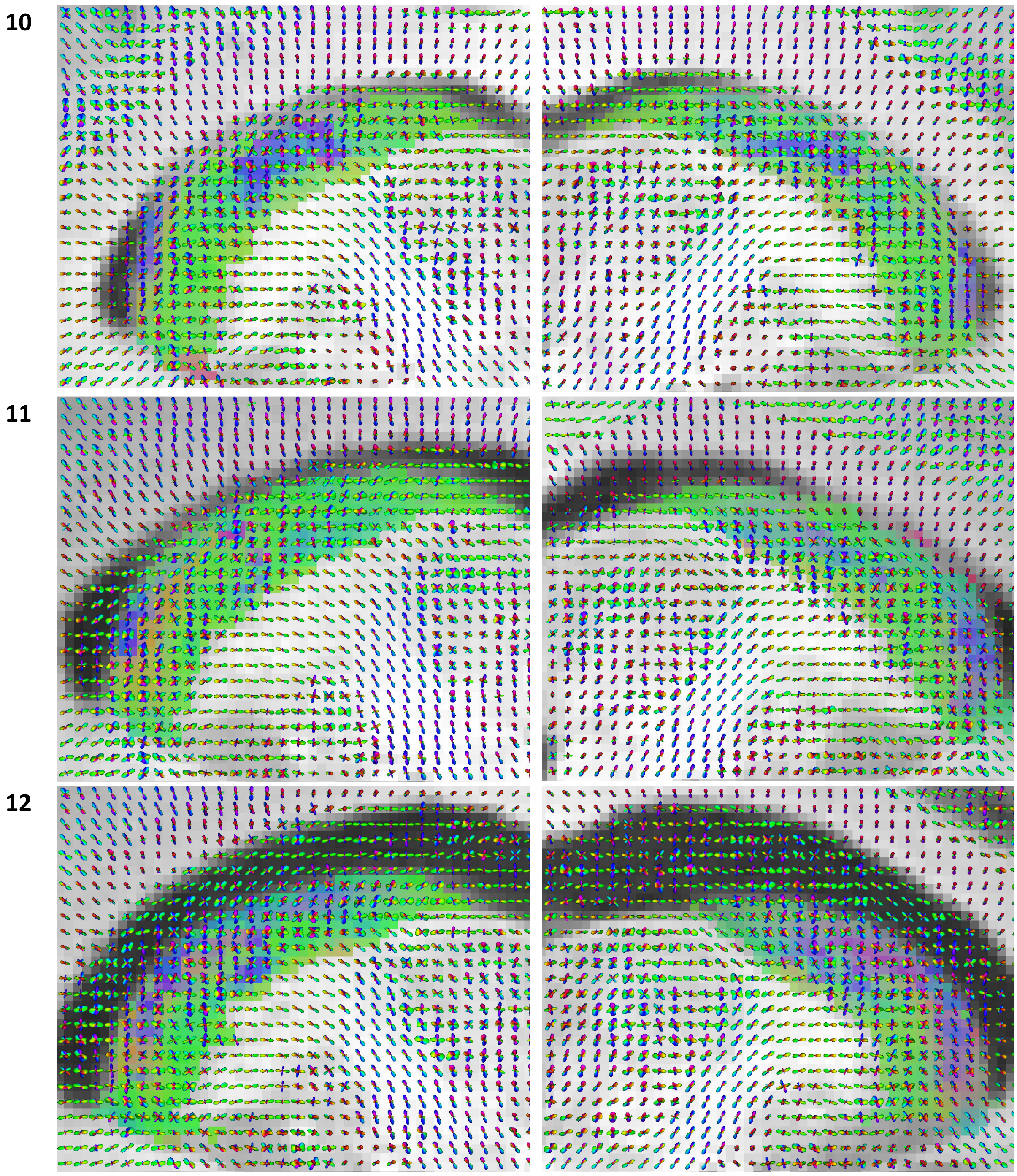
10
11
12

13 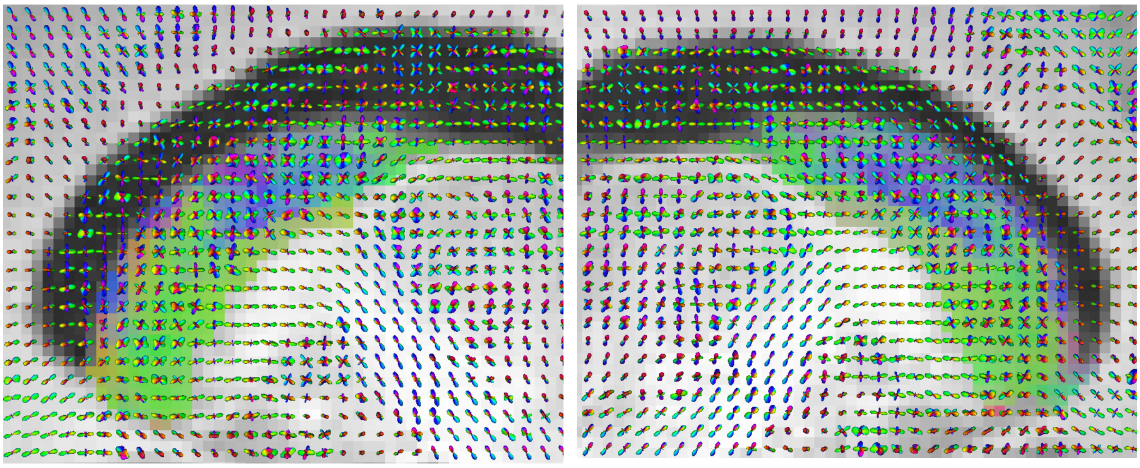

Figure S2: Complex fiber orientation structure visualized as orientation density functions overlaid to the direction-coded color image derived from diffusion tensor imaging (DTI). This sagittal images of all participants shows that the principle diffusion directions as defined by the diffusion tensors represent subtle but consistent imbalances within the complex fiber orientation profile of the caudate nucleus (CN) gray matter. While fibers running along the axis of the CN outweigh the fibers entering from the adjacent white matter of the internal capsule (IC) in the posterior portion, in the anterior portion, it seems that fibers entering from the IC dominate the diffusion profile. The middle portion with inferior-superior principal directions (blue area) is characterized by additional fibers restricted to the caudate and pointing in the direction of the external globus pallidus (GPe).
