# Supplementary material for: Beyond Cytoarchitectonics: The Internal and External Connectivity Structure of the Caudate Nucleus
Source: PLoS One. 2013 Jul 26;8(7):e70141. doi: 10.1371/journal.pone.0070141 (PMC3724823; doi:10.1371/journal.pone.0070141)
Supplement: Figure S3 — Three-dimensional interactive rendering of the fiber populations of the caudate (see Fig 5 for details). Click into the Figure to activate the interface. Left mouse click permits rotation, wheel of zooming. The 3D model was integrated in the portable document format (PDF) using SimLab Composer (SimLab Soft., Amman, Jordan) and requires the use of a compatible PDF reader (e.g. Adobe Reader 9). (PDF) [file pone.0070141.s003.pdf]

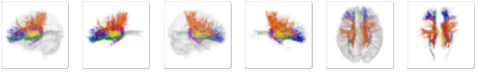

Figure 6: Three-dimensional interactive rendering of the fiber populations of the caudate (see Fig 5 for details). Click into the Figure to activate the interface. Left mouse click permits rotation, wheel of zooming. The 3D model was integrated in the portable document format (PDF) using SimLab Composer (SimLab Soft., Amman, Jordan) and requires the use of a compatible PDF reader (e.g. Adobe Reader 9).
